# Purification and Characterization of Antioxidant Peptides of *Pseudosciaena crocea* Protein Hydrolysates

**DOI:** 10.3390/molecules22010057

**Published:** 2016-12-30

**Authors:** Ningning Zhang, Chong Zhang, Yuanyuan Chen, Baodong Zheng

**Affiliations:** 1 College of Food Science, Fujian Agriculture and Forestry University, Fuzhou 350002, China; znnfst@163.com (N.Z.); cyyfst@163.com (Y.C.); 2 Fuzhou Municipal Finance Office, Fuzhou 350002, China; zhangchong2008cn@163.com

**Keywords:** *Pseudosciaena crocea*, antioxidant, peptide purification, chromatographic separation, amino acid sequence

## Abstract

Two peptides with antioxidant activity were isolated from *Pseudosciaena crocea* proteins. *Pseudosciaena crocea* muscle was hydrolyzed with neutral protease to obtain *Pseudosciaena crocea* protein hydrolysates (PCPH). After ultrafiltration through molecular weight cut-off membranes of 10, 5 and 3 kDa and assessment of free radical scavenging ability, the fraction (PCPH-IV) with the highest antioxidant activity was obtained. Several purification steps, i.e., ion exchange chromatography, gel filtration chromatography and reversed phase high performance liquid chromatography, were applied to further purify PCPH-IV. Two antioxidant peptides with the amino acid sequences Ser-Arg-Cys-His-Val and Pro-Glu-His-Trp were finally identified by LC-MS/MS.

## 1. Introduction

Free radicals are important signaling molecules that play key roles in gene expression, cell division and other normal physiological processes. However, excessive levels of free radicals in cells can cause lipid peroxidation, endogenous enzyme deactivation, mutation and loss of genetic information, and are thus believed to be responsible for causing several chronic diseases, including diabetes, cardiovascular diseases and cancers [1]. Therefore, it has been suggested that sufficient amounts of antioxidants need to be consumed to prevent oxidative stress caused by free radicals [2]. Recently, there has been increasing interest in natural antioxidants owing to their potential health benefits and minimal side effects compared with synthetic antioxidants [3]. Different kinds of antioxidant peptides have been isolated from cereal protein [3,4], fish protein [5,6,7,8], seed protein [9,10], egg white protein [11] and other food-borne proteins [12,13].

*Pseudosciaena crocea* is a maricultured fish species in China [14]. It is widely consumed because of its palatability and nutritional value [15]. However, approximately 40% of the protein-rich fish processing byproducts are discarded or used in lower-value products, such as fishmeal, fertilizer and animal feed [16]. Recently, many kinds of fish proteins and fish byproducts have been hydrolyzed to obtain antioxidant peptides, such as peptides from tuna backbone [17], hoki frame protein [5], tuna dark muscle [6], tilapia frame protein [7], salmon byproducts [2] and croceine croaker muscle [8]. These studies have indicated that fish proteins and byproducts can be used as a good source of antioxidant peptides. Hence, by using enzymatic hydrolysis, the utilization rate and economic value of fish proteins and byproducts can be improved.

In a previous study, we used different enzymes to prepare antioxidant peptides from *Pseudosciaena crocea* and demonstrated that peptides obtained by neutral protease digestion exhibited the strongest antioxidant activities. Although Chi et al. [8] have purified three antioxidant peptides, the isolation of peptides from *Pseudosciaena crocea* has not yet been fully explored. In particular, many of the peptides have not been identified. Therefore, the objectives of this study were to prepare *Pseudosciaena crocea* protein hydrolysates, evaluate their antioxidant properties and further purify and identify the antioxidant peptides.

## 2. Results and Discussion

### 2.1. Free Radical Scavenging Ability of Pseudosciaena crocea Protein Hydrolysates (PCPH) after Ultrafiltration

**Figure 1:**
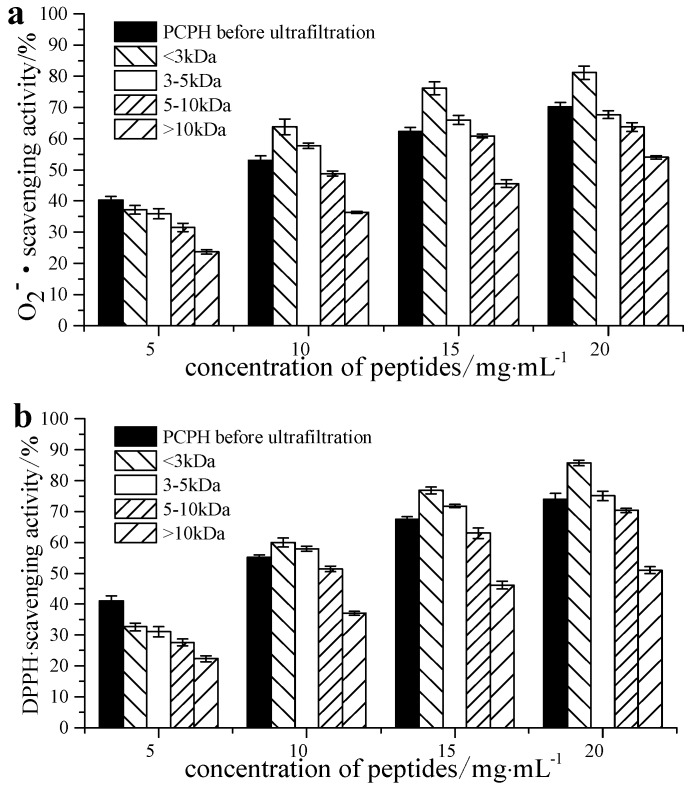
Free radical scavenging activity of fractions of PCPH at different concentrations: (**a**) O_2_^−^· scavenging activity; (**b**) DPPH scavenging activity.

Ultrafiltration is widely used in the preparation of biologically active peptides [18]. In the present study, PCPH were initially separated into four fractions, PCPH-I (MW > 10 kDa), PCPH-II (5 kDa < MW < 10 kDa), PCPH-III (3 kDa < MW < 5 kDa) and PCPH-IV (MW < 3 kDa), by ultrafiltration. All fractions were dissolved in distilled water at concentrations of 5, 10, 15 or 20 mg/mL. The free radical scavenging ability of samples of PCPH before ultrafiltration, PCPH-I, PCPH-II, PCPH-III and PCPH-IV at different concentrations, are shown in Figure 1. All hydrolysates showed significant antioxidant activity toward scavenging the different radical species tested. Increasing the concentration of hydrolysates caused a dose-dependent increase in antioxidant ability.

It was known that antioxidant activity is affected by the size and compositional changes of peptides [19] and low-molecular-weight peptides are more biologically active compared to their parent large polypeptides [20]. As shown in Figure 1, the radical scavenging ability of PCPH-IV (IC_50_ of 7.67 mg/mL for scavenging O_2_^−^∙, 7.68 mg/mL for DPPH) was the highest among all the PCPH fractions. Therefore, this fraction was used for further antioxidant assays and purification.

### 2.2. Effect of PCPH-IV on Antioxidant Enzyme Activities in HepG2 Cells

**Figure 2:**
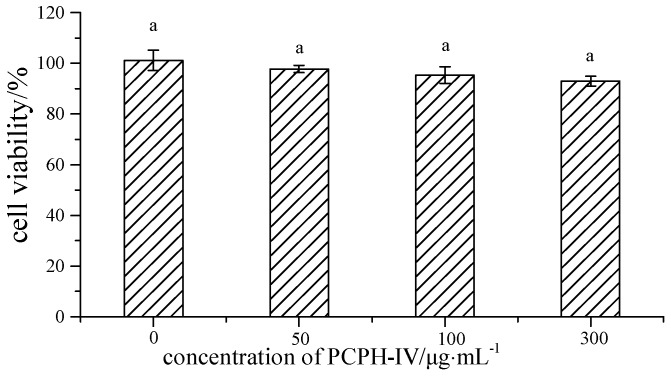
Effects of PCPH-IV on HepG2 cell viability. Bars labeled with the same letter are not significantly different (*p* > 0.05).

Human hepatoma cells are often used as a model for studying the mechanisms of protection against oxidative stress [21]. Hence, the effects of the PCPH-IV fraction on cellular antioxidative markers in the HepG2 cell line were studied to further investigate its antioxidant activity. First, a cell viability assay was performed to check whether PCPH-IV caused any cellular damage. As shown in Figure 2, the growth of HepG2 cells was slightly affected (*p* > 0.05) by PCPH-IV. However, PCPH-IV did not show cytotoxicity in HepG2 cells up to a concentration of 300 µg/mL (cell viability > 90%).

**Figure 3:**
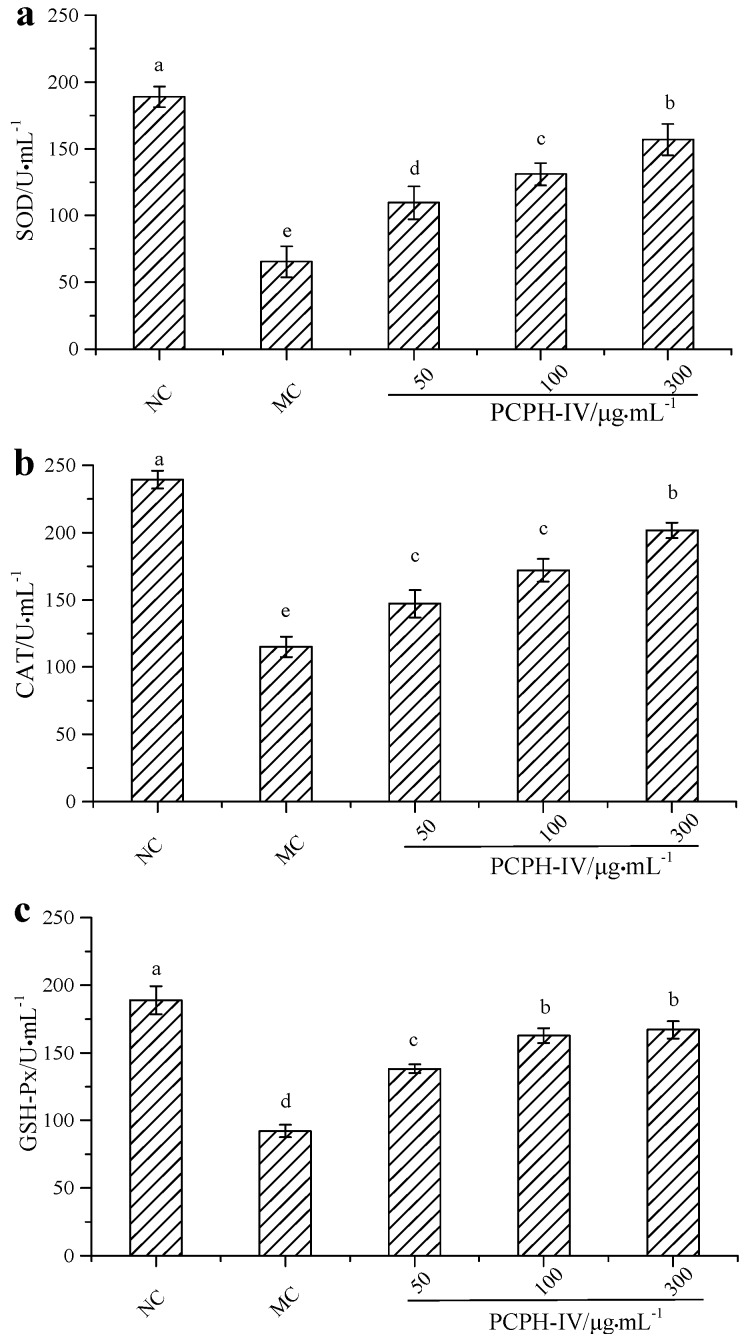
Effects of different concentrations of PCPH-IV on SOD (**a**); CAT (**b**); and GSH-Px (**c**) activities of HepG2 cells subjected to H_2_O_2_-induced oxidative stress (NC—untreated control. MC—treated with H_2_O_2_ alone). Different letters above the bars indicate significant differences at *p* < 0.05.

H_2_O_2_ can cause damage to intracellular biomacromolecules owing to its status as a strong oxidizer. It can also transform into other reactive oxygen species (ROS), such as hydroxyl radicals, superoxide anions and nitric oxide. Excess H_2_O_2_ is capable of inducing oxidative stress, apoptosis, and inflammation though the activation of the ERK/JNK MAPK and NF-κB pathways [22,23]. Antioxidant enzymes, such as SOD, CAT and GSH-Px, are considered the first line of the antioxidant defense system against ROS-mediated oxidative stress [24]. SOD is a key cellular antioxidant enzyme capable of converting superoxide anions to H_2_O_2_ and water and inhibiting the signaling process induced by superoxide anions [25]. Thus, the oxidative stress levels of cells can be evaluated by monitoring SOD activity. CAT and GSH-Px are able to degrade H_2_O_2_ into H_2_O and O_2_, preventing the formation of free radicals, such as hydroxyl radicals or peroxynitrite [26,27]. Therefore, to evaluate the protective effect of PCPH-IV on H_2_O_2_-treated HepG2 cells, antioxidant enzymes levels of SOD, CAT and GSH-Px were determined. As shown in Figure 3, compared with the untreated group, cells treated with 40 µmol/L H_2_O_2_ alone showed significantly reduced levels of SOD (188.91 ± 7.62 U/mL vs. 65.47 ± 11.59 U/mL), CAT (239.36 ± 6.53 U/mL vs. 115.23 ± 7.55 U/mL) and GSH-Px (188.89 ± 10.38 U/mL vs. 92.14 ± 4.59 U/mL). However, when pretreated with different concentrations of PCPH-IV, HepG2 exposure to H_2_O_2_ significantly up-regulated the levels of SOD, CAT and GSH-Px in a dose-dependent manner.

Previous studies have reported that plant bioactive compounds can act as indirect antioxidants by inducing up-regulation of antioxidant enzymes in cells and mice [21,24,28]. The present study demonstrates that PCPH-IV exhibited antioxidant activities in both non-biological assays and HepG2 cells. Hence, it is possible that PCPH-IV acts as a direct antioxidant by scavenging free radicals and as an indirect antioxidant by modulation of antioxidant enzymatic defenses [29].

### 2.3. Peptide Separation by Ion Exchange Chromatography and Sephadex G-15 Gel Chromatography

**Figure 4:**
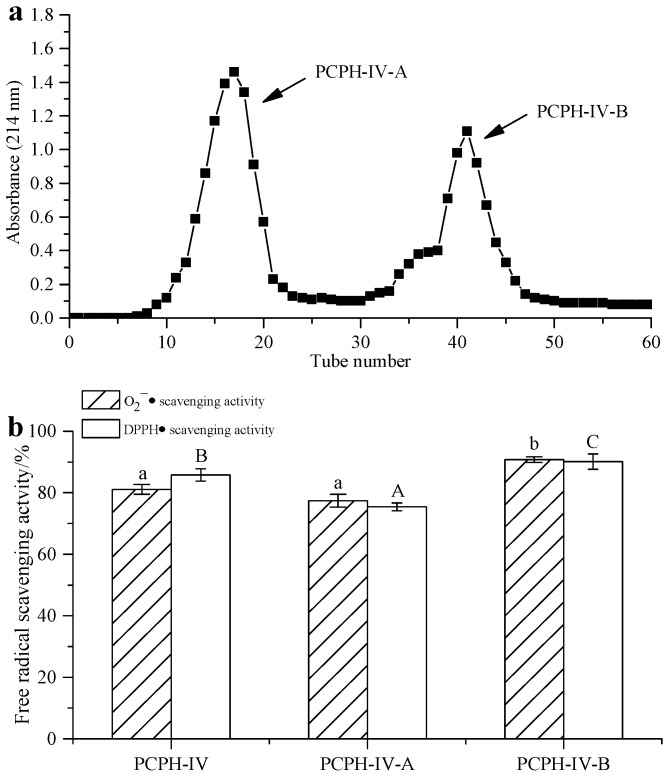
Separation of PCPH-IV by ion exchange chromatography: (**a**) chromatogram of PCPH-IV from cation exchange chromatography; (**b**) free radical scavenging activity of PCPH-IV fractions obtained from cation exchange chromatography. Identical same-case letters above the bars indicate no significant difference (*c* = 20 mg/mL, *p* > 0.05).

PCPH-IV was purified by cation exchange chromatography, yielding two different peaks (labeled as PCPH-IV-A and PCPH-IV-B, Figure 4a). PCPH-IV-B (with IC_50_ = 1.08 mg/mL for scavenging O_2_^−^, 0.85 mg/mL for DPPH) was found to exhibit stronger free radical scavenging activity than PCPH-IV-A (with IC_50_ = 13.34 mg/mL for scavenging O_2_^−^·, 9.27 mg/mL for DPPH, Figure 4b). Lyophilized PCPH-IV-B was further subjected to gel filtration chromatography.

**Figure 5:**
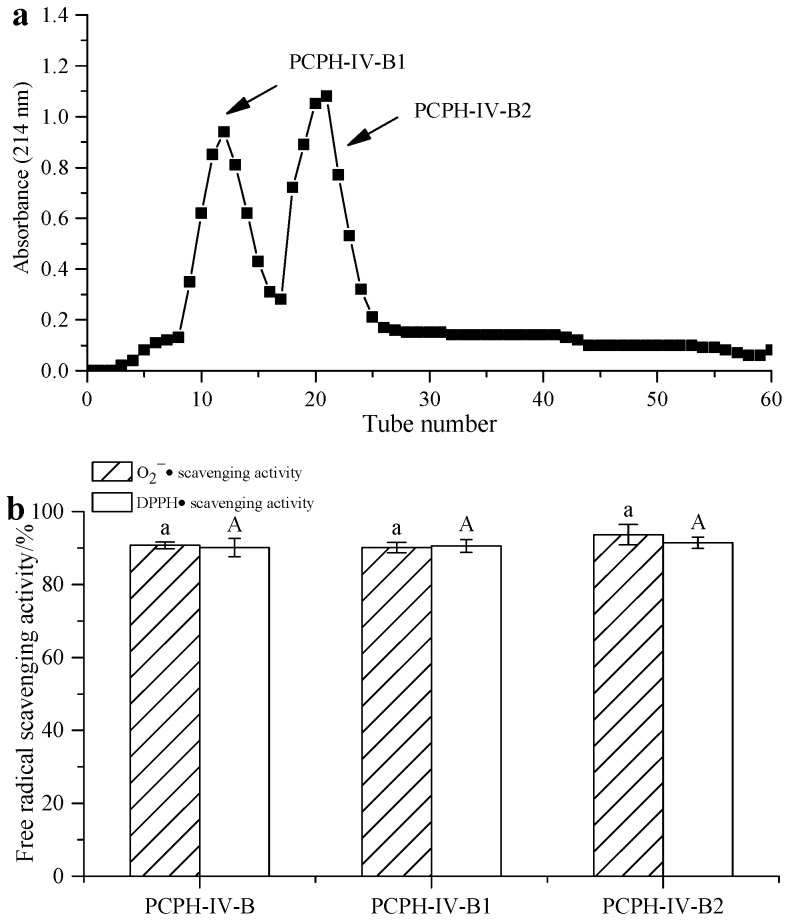
Separation of PCPH-IV-B by Sephadex G-15 gel chromatography: (**a**) chromatogram of PCPH-IV-B from Sephadex G-15 gel chromatography; (**b**) free radical scavenging activity of PCPH-IV-B fractions obtained from Sephadex G-15 gel chromatography. Identical same-case letters above the bars indicate no significant difference (*c* = 20 mg/mL, *p* > 0.05).

Gel filtration chromatography was applied to isolate the protein hydrolysates according to their molecular weight [30]. PCPH-IV-B was fractionated using Sephadex G-15 gel chromatography into two fractions (Figure 5a): the fraction with the shortest retention time (PCPH-IV-B1) corresponded to the largest-sized peptide fragments, whereas the fraction with the longest retention time (PCPH-IV-B2) corresponded to the smallest-sized peptide fragments. The antioxidant activity of both PCPH-IV-B1 (with IC_50_ = 0.93 mg/mL for scavenging O_2_^−^ , 0.77 mg/mL for DPPH) and PCPH-IV-B2 (with IC_50_ = 1.05 mg/mL for scavenging O_2_^−^ , 0.76 mg/mL for DPPH) was almost the same as that of PCPH-IV-B (Figure 5b). PCPH-IV-B1 and PCPH-IV-B2 were further purified by RP-HPLC.

### 2.4. Purification of Antioxidant Peptides by RP-HPLC

**Figure 6:**
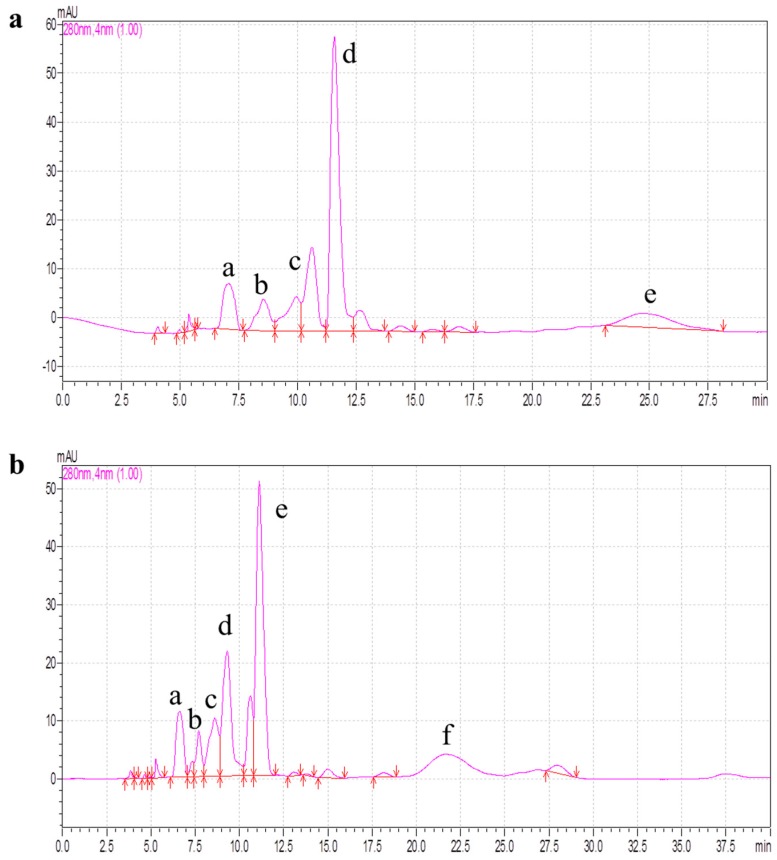
RP-HPLC chromatograms of (**a**) PCPH-IV-B1 (PCPH-IV-B1 was divided into five major fractions, labeled as a, b, c, d, e); and (**b**) PCPH-IV-B2 (PCPH-IV-B2 was divided into six major fractions, labeled as a, b, c, d, e, f).

RP-HPLC is widely used for the isolation and purification of peptides because of its high speed, high sensitivity and good reproducibility [3]. After gel filtration, PCPH-IV-B1 and PCPH-IV-B2, which showed higher antioxidant ability than the other fractions from PCPH-IV, were further separated by RP-HPLC. Figure 6 shows that the PCPH-IV-B1 was divided into five major fractions (labeled IV-B1-a, IV-B1-b, IV-B1-c, IV-B1-d, IV-B1-e) and PCPH-IV-B2 into six fractions (IV-B2-a, IV-B2-b, IV-B2-c, IV-B2-d, IV-B2-e, IV-B2-f). All these fractions were collected and the antioxidant activities evaluated. The results showed that IV-B1-d and IV-B2-e had higher antioxidant activity than the other fractions (IV-B1-a with IC_50_ = 0.68 mg/mL for scavenging O_2_^−^ , 0.80 mg/mL for DPPH, IV-B1-c 0.92 mg/mL for O_2_^−^ , 0.79 mg/mL for DPPH, IV-B2-a 0.37 mg/mL for O_2_^−^ , 0.55 mg/mL for DPPH, IV-B2-d 0.93 mg/mL for O_2_^−^ , 0.82 mg/mL for DPPH; the antioxidant activities of IV-B1-b, IV-B1-e, IV-B2-b, IV-B2-c, IV-B2-f were not detected because of their low contents). Comparing the results of PCPH-IV after ultrafiltration (with IC_50_ = 7.67 mg/mL for scavenging O_2_^−^ , 7.68 mg/mL for DPPH), the radical scavenging activities of IV-B1-d (0.17 mg/mL for O_2_^−^ , 0.14 mg/mL for DPPH) and IV-B2-e (0.16 mg/mL for O_2_^−^ , 0.24 mg/mL for DPPH) were significantly improved after RP-HPLC. Chi et al. [8] hydrolyzed *Pseudosciaena crocea* with papain and alcalase, and obtained three antioxidant peptides named PC-1, PC-2, PC-3. Both the scavenging free radical ability and the peptide sequences were significantly different from our data, which indicated that the type of protease is an important factor in the production of antioxidant peptides.

### 2.5. Identification of Amino Acid Sequence of Antioxidant Peptides

**Figure 7:**
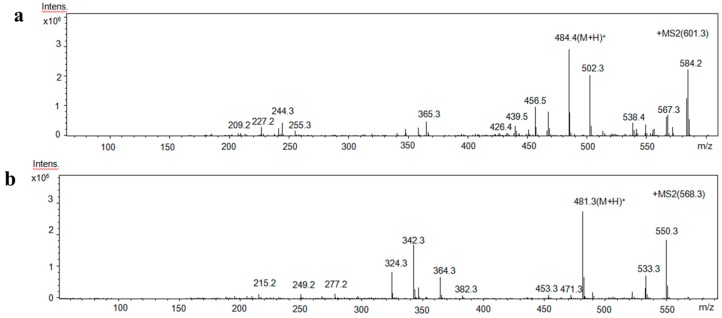
MS/MS spectra of (**a**) ion (*m*/*z* 601.3) from IV-B1-d; and (**b**) ion (*m*/*z* 568.3) from IV-B2-e.

Using LC-MS/MS, the two antioxidant peptides, IV-B1-d and IV-B2-e, were found to have the amino acid sequences Ser-Arg-Cys-His-Val and Pro-Glu-His-Trp, respectively (Figure 7), i.e., containing only four to five amino acids. This result is similar to a previous study, which reported that bioactive peptides are short peptides, usually consisting of two to 20 amino acids [31]. Morgan et al. [32] reported that the composition and position of amino acids in a peptide sequence are important for its bioactivity. The antioxidant activity of our purified peptides might be due to the presence of Val, Pro, Glu and Trp. A previous study has reported that aromatic residues such as Trp, Tyr and Phe can capture free radicals by providing protons [33]. By promoting interactions with free radicals, hydrophobic amino acids such as Val, Pro, Ala, Leu can improve the lipophilicity of peptides [3,19]. Other amino acids, including Trp, Glu, His, Cys, have also been reported to contribute to the antioxidant properties of peptides [2,34,35].

## 3. Materials and Methods

### 3.1. Materials

*Pseudosciaena crocea* was kindly provided by Fujian Fuding Seagull Fishing Food Co., Ltd. (Fuding, Fujian, China) without head, tail, skin, bone, internal organs and blood. The moisture, protein, fat and mineral contents of *Pseudosciaena crocea* were 69.1%, 17.5%, 11.1% and 1.1%, respectively. Neutral proteinase was purchased from Solarbio Science & Technology Co., Ltd., (Beijing, China). Dulbecco’s modified Eagle’s medium (DMEM), fetal bovine serum, gentamicin, penicillin G and streptomycin were purchased from Sigma-Aldrich Chemical Co., (St. Louis, MO, USA).

### 3.2. Preparation and Fractionation of Pseudosciaena crocea Protein Hydrolysates (PCPH)

PCPH were prepared using a method described by Zhang et al. [16]. The muscle of *Pseudosciaena crocea* was minced in a meat grinder (Yongkang Dili Industrial and Trading Co., Ltd., Zhejiang, China), and defatted with isopropanol using a sample:solvent ratio of 1:4 (*w*/*v*) at 60 °C. After 2 h defatting, the isopropanol was removed. The defatted samples (265.5 g) were homogenized in distilled water (1000 mL), and hydrolyzed with neutral protease using an enzyme:substrate ratio (E:S) of 1:25 after adjusting the pH of the mixture to 7.0. The digestion was performed in a water bath shaker to maintain a constant temperature (46 °C). After 7.2 h digestion, the hydrolysates were heated to 95 °C for 10 min to inactivate the protease, then centrifuged at 8000× *g* for 30 min and the supernatants collected.

PCPH were fractionated by ultrafiltration using membranes with a molecular weight cut-off (MWCO) of 3, 5 and 10 kDa (Vivaflow 200 Minimate, Sartorius, Gottingen, Germany). Four series of peptides were obtained: PCPH-I MW >10 kDa, PCPH-II 5 kDa < MW < 10 kDa, PCPH-III 3 kDa < MW < 5 kDa, PCPH-IV MW < 3 kDa. All the fractions were freeze-dried before conducting the antioxidant analyzes.

### 3.3. Free Radical Scavenging Activity 

Free radical scavenging activity was measured by two methods. Superoxide anion radical (O_2_^−^∙) scavenging ability was determined according to a method of Alashi et al. [36]. DPPH scavenging ability was measured using a method described by Siow et al. [37].

### 3.4. Assessment of Antioxidant Enzyme Activity in HepG2 Cells 

#### 3.4.1. Cell Culture

Human hepatoma cells (HepG2 cells, obtained from Fujian Medical University, Fuzhou, Fujian, China) were cultured and maintained in DMEM supplemented with 2.5% (*v*/*v*) fetal bovine serum, 50 mg/L gentamicin, 50 mg/L penicillin G and 50 mg/L streptomycin in a humidified atmosphere of 5% CO_2_ at 37 °C.

#### 3.4.2. Cell Viability

The fraction of PCPH showing the greatest potential to scavenge radicals after ultrafiltration was selected for cellular experiments. The effects of antioxidant peptides on the viability of HepG2 cells were determined by an MTT-based assay. Cells were seeded in a flat-bottomed 96-well plate at a density of 5.0 × 10^4^ cells/well, incubated for 24 h, then treated with different concentrations of antioxidant peptides (50, 100, 300 µg/mL). After 24 h of incubation, 20 μL MTT solution (5 mg/mL) was added to each well and the cells were incubated at 37 °C for 4 h. Afterwards, the supernatant was removed and 150 µL dimethyl sulfoxide was added to each well to dissolve formazan crystals. The absorbance was measured at 570 nm on a microplate reader. The results were expressed as percentages of viable cells in comparison to the control.

#### 3.4.3. Antioxidant Enzyme Activity Assays in H_2_O_2_ Challenged HepG2 Cells

HepG2 cells were seeded in a 96-well plate at a density of 5.0 × 10^4^ cells/well and incubated for 24 h, corresponding to 80%–90% confluency. Next, different concentrations of antioxidant peptides (50, 100, 300 µg/mL) were added to each well. After 4 h exposure to antioxidant peptides, 400 µmol/L H_2_O_2_ was added. After 24 h of incubation, the cells were washed with 1 mL phosphate-buffered saline (PBS) and harvested in an ice-cold cell lysis buffer. The cell debris was subjected to sonication and centrifugation at 12,000× *g* for 25 min and the supernatant was collected for analysis. The activities of superoxide dismutase (SOD), catalase (CAT) and glutathione peroxidase (GSH-Px) were measured with assay kits (product No. A001-1, A007-1, and A005, respectively; Nanjing Jiancheng Bioengineering Institute, Nanjing, China) according to the manufacturer’s instructions.

### 3.5. Purification of Antioxidant Peptides from PCPH

#### 3.5.1. Ion Exchange Chromatography

The series of peptides with the highest antioxidant activity after ultrafiltration (PCPH-IV) was dissolved in sodium acetate buffer (20 mM, pH 3.6) at a concentration of 40 mg/mL, and loaded onto a cation exchange column (25 mm × 500 mm, Shanghai Huxi Analysis Instrument Factory CO., Ltd., Shanghai, China). The column was equilibrated with the same buffer and then eluted with Tris-HCl butter (pH 7.2) at a constant flow rate of 0.8 mL/min. Fractions (4 mL) were collected and monitored at 214 nm. Each fraction was pooled, lyophilized and subjected to DPPH and O_2_^−^· scavenging assays.

#### 3.5.2. Gel Filtration Chromatography (GFC)

The peptide fraction showing the highest antioxidant activity after separation by ion exchange chromatography was further purified using gel filtration chromatography on a column (25 mm × 500 mm, Shanghai Huxi Analysis Instrument Factory CO., Ltd., Shanghai, China) packed with Sephadex G-15 (Biosharp Biological Technology Co., Ltd., Hefei, Anhui, China). The elution was carried out with distilled water at a constant flow rate of 0.8 mL/min. Each fraction (4 mL) was collected and monitored at 214 nm. All fractions were lyophilized prior to being analyzed in antioxidant assays.

#### 3.5.3. RP-HPLC

After separation by GFC, the fractions were further purified using reverse-phase high performance liquid chromatography (RP-HPLC). The lyophilized peptides were dissolved in distilled water at a concentration of 20 mg/mL and then injected into a Shimadzu LC-20A system (Shimadzu Corporation, Tokyo, Japan). The samples were eluted using 0.03% trifluoroacetic acid (TFA) in water (A) and 100% acetonitrile containing 0.03% TFA (B) at a flow rate of 0.8 mL/min. The following elution gradient was used: 0–10 min, linear gradient 0%–10% B; 10–20 min, linear gradient 10%–35% B; 20–55 min, linear gradient 35%–70% B. The fractions were detected at 280 nm, collected, freeze-dried and subjected to antioxidant assays.

### 3.6. Amino Acid Sequencing of Purified Peptides

The fraction with the strongest antioxidant ability after RP-HPLC purification was subjected to LC-MS/MS analysis using a LC/MSD Trap XCT system (Agilent Technologies, Santa Clara, CA, USA) according to a method described by Shen et al. [38]. Spectra were recorded in positive ion reflector mode with a mass/charge (*m*/*z*) range of 200–1000. Peptide sequencing was performed by processing the MS/MS spectra using BioTools (Version 3.0, Bruker Daltonics lnc., Karlsdorf-Neuthard, Germany) as well as manual calculation.

### 3.7. Statistical Analysis

All results were calculated from three replicates and expressed as mean ± standard deviation. Statistical analysis was performed using SPSS 19.0 software (Armonk, NY, USA). Data were analyzed using one-way analysis of variance (ANOVA). Differences were considered to be significant at *p* < 0.05.

## 4. Conclusions

In the present study, two peptides with sequences Ser-Arg-Cys-His-Val and Pro-Glu-His-Trp were isolated and identified from PCPH. The results suggest that *Pseudosciaena crocea* has a potential value as a functional food ingredient. For further development as a novel antioxidative ingredient, the in vivo effects of these peptides should be investigated.

## References

[1] Soobrattee M.A., Neergheen V.S., Luximon-Ramma A., Aruoma O.L., Bahourun T. (2005). Phenolics as potential antioxidant therapeutic agents: Mechanism and actions. Mutat. Res..

[2] Ahn C.B., Kim J.B., Je J.Y. (2014). Purification and antioxidant properties of ocapeptide from salmon byproduct protein hydrolysate by gastrointestinal digestion. Food Chem..

[3] Agrawal H., Joshi R., Gupta M. (2016). Isolation, purification and characterization of antioxidative peptide of pearl millet (*Pennisetum glaucum*) protein hydrolysate. Food Chem..

[4] Wang J.S., Zhao M.M., Zhao Q.Z., Jiang Y.M. (2007). Antioxidant properties of papain hydrolysates of wheat gluten in different oxidation systems. Food Chem..

[5] Kim S.Y., Je J.Y., Kim S.K. (2007). Purification and characterization of antioxidant peptide from hoki (*Johnius belengerii*) frame protein by gastrointestinal digestion. J. Nutr. Biochem..

[6] Je J.Y., Qian Z.J., Lee S.H., Byun H.G., Kim S.K. (2008). Purification and antioxidant properties of bigeye tuna (*Thunnus obesus*) dark muscle peptide on free radical-mediated oxidative systems. J. Med. Food.

[7] Fan J., He J., Zhuang Y., Sun L. (2012). Purification and identification of antioxidant peptides from enzymatic hydrolysates of Tilapia (*Oreochromis niloticus*) frame protein. Molecules.

[8] Chi C.F., Hu F.Y., Wang B., Ren X.J., Deng S.G., Wu C.W. (2015). Purification and characterization of three antioxidant peptides from protein hydrolyzate of croceine croaker (*Pseudosciaena crocea*) muscle. Food Chem..

[9] Xue Z., Yu W., Liu Z., Wu M., Kou X., Wang J. (2009). Preparation and antioxidative properties of a Rapeseed (*Brassica napus*) protein hydrolysate and three peptide fractions. J. Agric. Food Chem..

[10] Ngoh Y.Y., Gan C.Y. (2016). Enzyme-assisted extraction and identification of antioxidative and α-amylase inhibitory peptides from Pinto beans (*Phaseolus vulgaris* cv. Pinto). Food Chem..

[11] Liu J., Yan J., Lin S., Jones G.S., Feng C. (2015). Purification and identification of novel antioxidant peptides from egg white protein and their antioxidant activities. Food Chem..

[12] Nimalaratne C., Bandara N., Wu J. (2015). Purification and characterization of antioxidant peptides from enzymatically hydrolyzed chicken egg white. Food Chem..

[13] Ohata M., Uchida S., Zhou L., Arihara K. (2016). Antioxidant activity of fermented meat sauce and isolation of an associated antioxidant peptide. Food Chem..

[14] Liu J.F., Han K.H. (2011). Current development situation and countermeasure of large yellow crocker industry in China. J. Fujian Fish..

[15] Li T., Hu W., Li J., Zhang X., Zhu J., Li X. (2012). Coating effects of tea polyphenol and rosemary extract combined with chitosan on the storage quality of large yellow croaker (*Pseudosciaena crocea*). Food Control.

[16] Zhang C., Zhang N., Li Z., Tian Y., Zhang L., Zheng B. (2016). Stability of antioxidant peptides prepared from the large yellow croaker (*Pseudosciaena crocea*). Curr. Top. Nutraceuticals Res..

[17] Je J.Y., Qian Z.J., Byun H.G., Kim S.K. (2007). Purification and characterization of an antioxidant peptide obtained from tuna backbone protein by enzymatic hydrolysis. Process Biochem..

[18] Moure A. (2006). Antioxidant properties of ultrafiltration-recovered soy protein fractions from industrial effluents and their hydrolysates. Process Biochem..

[19] Kou X., Gao J., Xue Z., Zhang Z., Wang H., Wang X. (2013). Purification and identification of antioxidant peptides from chickpea (*Cicer arietinum* L.) albumin hydrolysates. LWT Food Sci. Technol..

[20] Chen C., Chi Y.J., Zhao M.Y., Lv L. (2012). Purification and identification of antioxidant peptides from egg white protein hydrolysate. Amino Acids.

[21] Sowndhararajan K., Hong S., Jhoo J.-W., Kim S., Chin N.L. (2015). Effect of acetone extract from stem bark of Acacia species (*A. dealbata*, *A. ferruginea* and *A. leucophloea*) on antioxidant enzymes status in hydrogen peroxide-induced HepG2 cells. Saudi J. Biol. Sci..

[22] Wang C., Nie X., Zhang Y., Li T., Mao J., Liu X., Gu Y., Shi J., Xiao J., Wan C. (2015). Reactive oxygen species mediate nitric oxide production through ERK/JNK MAPK signaling in HAPI microglia after PFOS exposure. Toxicol. Appl. Pharm..

[23] Cao Y.J., Zhang Y.M., Qi J.P., Liu R., Zhang H., He L.C. (2015). Ferulic acid inhibits H_2_O_2_-induced oxidative stress and inflammation in rat vascular smooth muscle cells via inhibition of the NADPH oxidase and NF-κB pathway. Int. Immunopharmacol..

[24] Bak M.J., Jun M., Jeong W.S. (2012). Antioxidant and hepatoprotetive effects of the red ginseng essential oil in H_2_O_2_-treated HepG2 cells and CCl4-treated mice. Int. J. Mol. Sci..

[25] Halliwell B. (1994). Free radicals and antioxidants: A personal view. Nutr. Rev..

[26] Valéry A., Romuald C., Dragoslav M., Pascal C., Abderrahim L. (2007). Reactive oxygen species and superoxide dismutases: Role in joint diseases. Joint Bone Spine.

[27] Zhong W., Oberley L.W., Oberley T.D., Yan T., Domann F.E., St Clair D.K. (1996). Inhibition of cell growth and sensitization to oxidative damage by overexpression of manganese superoxide dismutase in rat glioma cells. Cell Growth Differ..

[28] Carvalho A.C., Franklin G., Dias A.C.P., Lima C.F. (2014). Methanolic extract of *Hypericum perforatum* cells elicited with *Agrobacterium tumefaciens* provides protection against oxidative stress induced in human HepG2 cells. Ind. Crop. Prod..

[29] Quéguineur B., Goya L., Ramos S., Martín M.A., Mateos R., Bravo L. (2012). Phloroglucinol: Antioxidant properties and effects on cellular oxidative markers in human HepG2 cell line. Food Chem. Toxicol..

[30] You L.J., Zhao M.M., Regenstein J.M., Ren J. (2010). Purification and identification of antioxidative peptides from loach (*Misgurnus anguillicaudatus*) protein hydrolysate by consecutive chromatography and electrospray ionization-mass spectrometry. Food Res. Int..

[31] Korhonen H., Pihlanto A. (2006). Bioactive peptides: Production and functionality. Int. Dairy J..

[32] Morgan P.E., Pattison D.I., Davies M.J. (2012). Quantification of hydroxyl radical-derived oxidation products in peptides containing glycine, alanine, valine and proline. Free Radic. Biol. Med..

[33] Huang D., Ou B., Prior R.L. (2005). The chemistry behind antioxidant capacity. J. Agric. Food Chem..

[34] Saiga A., Tanabe S., Nishmura T. (2003). Antioxidant activity of peptides obtained from porcine myofibrillar proteins by protease treatment. J. Agric. Food Chem..

[35] Zhu C.Z., Zhang W.G., Zhou G.H., Xu X.L., Kang Z.L., Yin Y. (2013). Isolation and identification of antioxidant peptides from Jinhua Ham. J. Agric. Food Chem..

[36] Alashi A.M., Blanchard C.L., Mailer R.J., Agboola S.O., Mawson A.J., He R., Girgih A., Aluko R.E. (2014). Antioxidant properties of Australian canola meal protein hydrolysates. Food Chem..

[37] Siow H.L., Gan C.Y. (2013). Extraction of antioxidative and antihypertensive bioactive peptides from *Parkia speciosa* seeds. Food Chem..

[38] Shen S., Chahal B., Majumder K., You S.J., Wu J. (2010). Identification of novel antioxidative peptides derived from a thermolytic hydrolysate of ovotransferrin by LC-Ms/MS. J. Agric. Food Chem..

